# Supplementary material for: From sequence to enzyme mechanism using multi-label machine learning
Source: BMC Bioinformatics. 2014 May 19;15:150. doi: 10.1186/1471-2105-15-150 (PMC4229970; doi:10.1186/1471-2105-15-150)
Supplement: Additional file 2 — Java code of ml2db. Additional file ml2db_code.tar.gz contains the Java source code to run the multi-label machine learning experiments and save the results to database. The code’s Javadoc is included. [file 1471-2105-15-150-S2.zip › additional file 2/ml2db/ml2db/doc/mulan/classifier/neural/package-tree.html]

mulan.classifier.neural Class Hierarchy


JavaScript is disabled on your browser.


- Overview
- Package
- Class
- Use
- Tree
- Deprecated
- Index
- Help

- Prev
- Next

- Frames
- No Frames

- All Classes

# Hierarchy For Package mulan.classifier.neural

Package Hierarchies:

- All Packages

## Class Hierarchy

- java.lang.Object
  - mulan.classifier.neural.BPMLLAlgorithm
  - mulan.classifier.neural.DataPair
  - mulan.classifier.neural.MMPUpdateRuleBase (implements mulan.classifier.neural.ModelUpdateRule)
    - mulan.classifier.neural.MMPMaxUpdateRule
    - mulan.classifier.neural.MMPRandomizedUpdateRule
    - mulan.classifier.neural.MMPUniformUpdateRule
  - mulan.classifier.MultiLabelLearnerBase (implements mulan.classifier.MultiLabelLearner, java.io.Serializable, weka.core.TechnicalInformationHandler)
    - mulan.classifier.neural.BPMLL
    - mulan.classifier.neural.MMPLearner
  - mulan.classifier.neural.NormalizationFilter (implements java.io.Serializable)
  - mulan.classifier.neural.ThresholdFunction (implements java.io.Serializable)

## Interface Hierarchy

- mulan.classifier.neural.ModelUpdateRule

## Enum Hierarchy

- java.lang.Object
  - java.lang.Enum<E> (implements java.lang.Comparable<T>, java.io.Serializable)
    - mulan.classifier.neural.MMPUpdateRuleType

- Overview
- Package
- Class
- Use
- Tree
- Deprecated
- Index
- Help

- Prev
- Next

- Frames
- No Frames

- All Classes
